# jmBIG: enhancing dynamic risk prediction and personalized medicine through joint modeling of longitudinal and survival data in big routinely collected data

**DOI:** 10.1186/s12874-024-02289-0

**Published:** 2024-08-06

**Authors:** Atanu Bhattacharjee, Bhrigu Kumar Rajbongshi, Gajendra K. Vishwakarma

**Affiliations:** 1 Population Health and Genomics, University of Dundee, Dundee, UK; 2 Department of Mathematics and Computing, Indian Institute of Technology-Dhanbad, Dhanbad, India

**Keywords:** Bayesian, Longitudinal, Survival

## Abstract

We have introduced the R package jmBIG to facilitate the analysis of large healthcare datasets and the development of predictive models. This package provides a comprehensive set of tools and functions specifically designed for the joint modelling of longitudinal and survival data in the context of big data analytics. The jmBIG package offers efficient and scalable implementations of joint modelling algorithms, allowing for integrating large-scale healthcare datasets.

By utilizing the capabilities of jmBIG, researchers and analysts can effectively handle the challenges associated with big healthcare data, such as high dimensionality and complex relationships between multiple outcomes.

With the support of jmBIG, analysts can seamlessly fit Bayesian joint models, generate predictions, and evaluate the performance of the models. The package incorporates cutting-edge methodologies and harnesses the computational capabilities of parallel computing to accelerate the analysis of large-scale healthcare datasets significantly. In summary, jmBIG empowers researchers to gain deeper insights into disease progression and treatment response, fostering evidence-based decision-making and paving the way for personalized healthcare interventions that can positively impact patient outcomes on a larger scale.

## Introduction

Prognostic tools are essential for physicians to make informed decisions about patient care. Joint models are a promising new prognostic tool, but it is essential to assess their predictive performance before they can be widely adopted [1, 2]. Prediction models are widely utilized in medicine, typically employing static inputs such as values obtained during clinic visits or hospital admissions. However, relying solely on values at a single point fails to account for the changing nature of covariate profiles over time and how these profiles influence the likelihood of an event. More intuitively, doctors update their prognoses by considering changes in biomarkers and the clinical status of their patients. Hence, the most effective prediction approach should employ a methodology that adequately incorporates all available changes in the predicted variables. While there have been statistical advancements in joint modelling and increased availability of longitudinal clinical measures, such as electronic medical records, the utilization of these innovations for adaptive individualized prediction remains limited in clinical research [3, 4].

One potential criticism of these models relates to the computational burden they impose. Additionally, despite various extensions discussed in the statistical literature, clinical research predominantly relies on the fundamental joint model. The computational burden of joint models can be a significant challenge, especially for large datasets.

This article aims to acquaint readers with dynamic risk predictions using extensive electronic health record (EHR) datasets, guiding them towards additional and more advanced resources such as theoretical concepts and practical code. We also emphasize the importance of measures to evaluate predictive performance and explore potential extensions that can enhance the accuracy of predictions.

Our recently developed R package provides access to simulated data that closely emulates real-world clinical scenarios. This dataset serves as a demonstrative example of the application of our approach. Automating big data analysis, specifically through joint longitudinal and survival modelling, can enhance patient outcomes and promote population health. This methodology enables the simultaneous analysis of longitudinal and survival data, resulting in precise and individualized predictions. Automation addresses the challenges of extensive and intricate datasets, revolutionizing healthcare analytics and improving patient care. Predictive models for time-to-event outcomes in medicine have become increasingly common. These models use longitudinal data from EHRs, patient registries, and established cohorts to develop dynamic prediction models that can be updated as new information becomes available. For example, dynamic prediction models have been used to predict 10-year cardiovascular disease risk based on EHRs [5], survival for people with cystic fibrosis using registry data [6], intervention-free survival for patients with aortic stenosis based on a cohort [3], and survival based on breast cancer recurrence data in a French cohort [7].

The development of predictive models for time-to-event outcomes in healthcare has the potential to revolutionize patient care. By identifying individuals at elevated risk for various health outcomes, healthcare providers can intervene early and provide targeted interventions to improve patient outcomes. Furthermore, big data and predictive models can help healthcare systems optimize resource allocation and improve population health. However, manual analysis of such a large dataset can be challenging and time-consuming, hence the need for automation.

Predictive modelling is a critical application of big data in healthcare, where longitudinal data from primary care records can be used to develop dynamic models for predicting various health outcomes. These models can help healthcare providers identify patients at high risk of developing specific conditions, allowing for early intervention and targeted treatment strategies. Developing predictive models is beneficial for time-to-event outcomes such as disease onset, recurrence, and mortality. Joint longitudinal and survival modelling is a specific technique used in predictive modelling that allows for the simultaneous analysis of longitudinal and survival data. This technique can predict the time-to-event outcome while accounting for the effect of longitudinal factors such as demographic characteristics, medical history, and treatment history. Joint modelling can provide more accurate and personalized predictions, allowing healthcare providers to intervene early and provide targeted treatments. Joint modelling on primary care big data can significantly improve patient outcomes and population health, making it an essential area of research in healthcare analytics. This work aims to present the automation of big data analysis using joint longitudinal and survival modelling. This approach allows for the simultaneous analysis of longitudinal and survival data to develop dynamic predictive models for time-to-event outcomes in healthcare. The automation of this process is critical due to the large and complex nature of big data, which can be challenging to analyze manually. By automating the analysis, healthcare providers can obtain more accurate and personalized predictions, improving patient outcomes and population health. Overall, this work aims to demonstrate the potential of joint longitudinal and survival modelling in automating big data analysis in healthcare and its role in improving patient care.

Longitudinal data are collected repeatedly over time for the same individuals. Time-to-event data record the time until an event of interest occurs, such as death or disease progression. Joint longitudinal-survival models are statistical models that can be used to analyze data, including longitudinal and time-to-event outcomes.

## Theoretical framework

Electronic health records (EHRs) are digital versions of paper medical records. They contain vast data about patients, including demographics, medications, allergies, immunizations, lab results, and imaging studies. This data can be used to improve care quality and coordination, but it also presents several challenges.

One challenge is the sheer volume of data. EHRs can contain terabytes or even petabytes of data, which can be difficult to manage and analyze. Another challenge is the heterogeneity of the data. EHR data can be in a variety of formats, and it can be challenging to integrate data from different sources.

Computational challenges also exist. EHR data can be computationally expensive to analyze, and it can be challenging to develop models that can accurately predict patient outcomes.

Despite these challenges, EHR data can be valuable for research and clinical decision-making. By developing new methods for managing, analyzing, and interpreting EHR data, researchers and clinicians can improve the quality of care and patient health.

Joint longitudinal-survival models have several advantages over separate longitudinal and time-to-event data models.

Accounting for dependence: Joint models can account for the dependence between the two data types. This is important because the longitudinal data can provide information about the risk of the event, and the time-to-event data can provide information about the course of the disease. For example, if a longitudinal outcome measures disease severity, then the risk of an event may be higher for individuals with more severe disease. Estimating treatment effects: Joint models can be used to estimate the effects of treatments or interventions on both the longitudinal and time-to-event outcomes. This can be done by including treatment as a covariate in the model. For example, a joint model could be used to estimate the effect of a new drug on both the progression of disease and the risk of death. In addition to these advantages, joint longitudinal-survival models can also identify prognostic factors, predict risk, and characterize disease course.

The bivariate proportional hazards model assumes a linear relationship, while the joint model for repeated measures and event occurrence is more flexible.

One difficulty is that the models can be computationally intensive, especially for large datasets. The models must account for the correlation between the longitudinal and time-to-event data.

Another difficulty is that the models can be sensitive to the choice of the model parameters. This means that the analysis results can vary depending on the values of the chosen parameters.

The effect of a treatment or intervention on both the longitudinal and time-to-event outcomes. The association between two longitudinal outcomes. The risk of an event over time. Despite the challenges of estimating these models, they can be a valuable tool for research.

Let $Y_i(t)$ denote the longitudinal outcome for individual *i* at time *t*, and let $T_i$ denote the time to the event for individual *i*. The joint longitudinal-survival model is for the joint distribution of $(Y_i(t), T_i)$.

One common type of joint longitudinal-survival model is the bivariate proportional hazards model. This model assumes that the hazard of the event for individual *i* at time *t* is given by

1 $$\begin{aligned} h_i(t) = h_0(t) \exp (x_i' \beta + u_i(t)) \end{aligned}$$

where $h_0(t)$ is an unspecified baseline hazard function, $x_i$ is a vector of covariates, $\beta$ is a vector of parameters, and $u_i(t)$ is a function of the longitudinal data.

In other words, the bivariate proportional hazards model assumes that the hazard of the event is proportional to a function of the longitudinal data plus a function of the covariates. This function of the longitudinal data can capture the association between the longitudinal and time-to-event outcomes.

For example, if the longitudinal outcome measures disease severity, then the hazard of the event may be higher for individuals with more severe disease.

Another common joint longitudinal-survival model is the joint model for repeated measures and event occurrence. This model allows for different types of dependence between the longitudinal and time-to-event data. For example, the model can be specified to allow for the longitudinal data to affect the hazard of the event or the time to the event to affect the longitudinal data.

The estimation of joint longitudinal-survival models can be challenging. One challenge is that the models may be computationally intensive, especially for large datasets. Another challenge is that the models may be sensitive to the choice of the model parameters [8].

Several different methods can be used to estimate joint longitudinal-survival models. One standard method is the maximum likelihood method. This method maximises the likelihood of the observed data under the model. The Kaplan-Meier estimator is a non-parametric method used to estimate the survival function from time-to-event data, particularly when the data may be censored. Given a set of observed survival times $t_1, t_2, ..., t_n$ and corresponding censoring indicators $C_1, C_2, ..., C_n$, where $C_i = 1$ if the event occurred at time $t_i$ and $C_i = 0$ if the event was censored, the Kaplan-Meier estimator of the survival function *S*(*t*) at time *t* is calculated as follows:

2 $$\begin{aligned} \hat{S}(t)=\prod _{t_{i}<t}\frac{n_{i}-d_{i}}{n_{i}} \end{aligned}$$

where $n_i$ is the number of individuals at risk just before time $t_i$, and $d_i$ is the number of events (deaths) at time $t_i$.

The likelihood function for a bivariate proportional hazards model [9] is given by

3 $$\begin{aligned} L(\beta , \theta ) = \prod _{i=1}^n \left[ h_0(T_i) \exp (x_i' \beta + u_i(T_i)) \right] ^{I(T_i < \tau )} \prod _{i=1}^n f(Y_i(t), t) \end{aligned}$$

where *n* is the number of individuals, $I(T_i < \tau )$ is an indicator function that is equal to 1 if individual *i* has experienced the event, and 0 otherwise, $\tau$ is the maximum follow-up time, $f(Y_i(t), t)$ is the joint distribution of the longitudinal data, and $\theta$ is a vector of parameters that specifies the distribution of the longitudinal data.

The maximum likelihood estimates of $\beta$ and $\theta$ can be obtained by maximizing the likelihood function using an iterative optimization algorithm [10].

Another method that can be used to estimate joint longitudinal-survival models is the Bayesian method. This method specifies a prior distribution for the model parameters and then uses Bayes’ theorem to update the prior distribution with the observed data.

The Bayesian posterior distribution for the model parameters is given by

4 $$\begin{aligned} p(\beta , \theta | y, t, d) \propto L(\beta , \theta ) p(\beta , \theta ) \end{aligned}$$

where *y* is the vector of longitudinal data, *t* is the vector of event times, and *d* is the vector of censoring indicators.

The Bayesian estimates of $\beta$ and $\theta$ can be obtained by sampling from the posterior distribution using a Markov chain Monte Carlo (MCMC) algorithm [11].

Consider a dataset $D_{n}={T_{i},\delta _{i}, y_{i};i=1,....,n}$ sampled from a target population. Each subject *i* has a true event time denoted as $T_{i}$ and a censoring time denoted as $C_{i}$. The observed event time $T_{i}$ is defined as the minimum between $T_{i}$ and $C_{i}$. The event indicator $\delta _{i}$ is defined as 1 if $T_{i} \le C_{i}$, and 0 otherwise. Additionally, the subject *i* has a longitudinal response vector $y_{i}$ of size $n_{i} \times 1$, where each element $y_{il}$ corresponds to the longitudinal outcome at time point $t_{il}$.

We propose a generalized linear mixed-effects model to accommodate various longitudinal response types. This model assumes that the conditional distribution of $y_{i}$ given the random effects vector $b_{i}$ follows an exponential family distribution. The linear predictor $\eta _i(t)$ is given by the equation:

5 $$\begin{aligned} g(E{y_i(t) | b_i}) = \eta _i(t) = x_i^{T}(t) \beta + z_i^{T}(t) b_i, \end{aligned}$$

where *g *(.) represents a known one-to-one monotonic link function, $y_{i}$ represents the longitudinal outcome at time *t* for subject *i*, $x_{i}(t)$ and $z_{i}(t)$ represent the time-dependent design vectors for the fixed effects $\beta$ and random effects $b_{i}$, respectively.

The random effects are assumed to follow either a multivariate normal distribution with zero mean and covariance matrix *D* or a multivariate Student ’s-t distribution with zero mean, covariance matrix *D*, and *df* degrees of freedom.

For the survival process, the event risk depends on a function of the subject-specific linear predictor $\eta _{i}(t)$. Specifically, the hazard function $h_i(t | H_i(t), w_i(t))$ is defined as:

6 $$\begin{aligned} h_i(t | H_i(t), w_i(t)) = \lim _{\Delta t \rightarrow 0} \frac{1}{\Delta t} P(t \le T_i^* < t + \Delta t | T_i^* \ge t, H_i(t), w_i(t)), \end{aligned}$$

7 $$\begin{aligned} h_i(t | H_i(t), w_i(t)) = h_0(t) \exp (\gamma w_i(t) + f(H_i(t), b_i, \alpha )), \end{aligned}$$

where $h_i(t | H_i(t), w_i(t))$ represents the hazard function for subject *i* at time *t*. Here, $H_i(t)$ denotes the subject’s event history up to time *t*, $w_i(t)$ represents additional time-dependent covariates, $h_0(t)$ represents the baseline hazard function, $\gamma$ represents the coefficient associated with $w_i(t)$, and $f(H_i(t), b_i, \alpha )$ represents a function of the subject’s event history, random effects $b_i$, and other parameters.

Where $H_{i}(t)=\{\eta _{i}(s)), 0 \le s < t\}$ denotes the history of the underlying longitudinal process up to $h_{0}(t)$ denotes the baseline hazard function, $w_{i}(t)$ is a vector of exogenous, possibly time-varying, covariates with corresponding regression coefficients $\gamma$. Parameter vector $>\alpha$ quantifies the association between features of the marker process up to time *t* and the hazard of an event at the same time point. To complete the specification of the survival process, we need to make appropriate assumptions for the baseline hazard function $h_{0}(t)$. We use a B-splines approach to model this function while still allowing for flexibility. In particular, the logarithm of the baseline hazard function is expressed as

8 $$\begin{aligned} \log h_0(t) = \gamma _0^0 + \sum _{q = 1}^Q \gamma _0^q B_q(t, v) \end{aligned}$$

where $B_{q}(t, v)$ denotes the *q* th basis function of a B-spline with knots $v_{1},...., v_{Q}$ and $\gamma _0^{q}$ the vector of spline coefficients. Increasing the number of knots *Q* increases the flexibility in approximating $\text{ log } h_{0}(t)$; however, we should balance bias and variance and avoid over-use code with caution.

## R packages for joint modeling data analysis

**Fig. 1:**
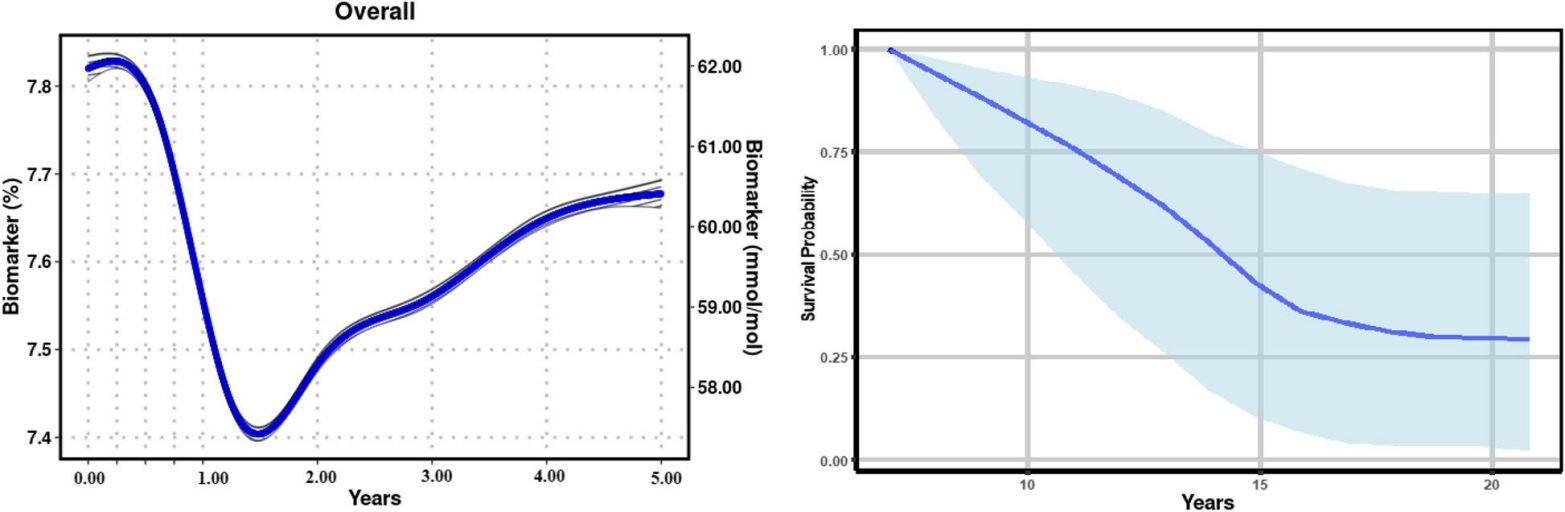
Using the jmBig package for longitudinal and survival predictions, the left-hand side image displays the predicted values of the Biomarker obtained through longitudinal modelling, while the right-hand side image presents the predicted survival probability of those individuals

**JMbayes2** is a robust R package for simultaneous longitudinal and survival data analysis. It offers comprehensive tools for developing dynamic predictive models that account for complex relationships between these outcomes. Bayesian methods estimate model parameters, providing flexibility and accuracy in complex data structures. Additionally, **JMbayes2** includes visualization and diagnostic tools for model evaluation and selection, making it a valuable tool for analyzing big healthcare data and improving patient outcomes (Fig. 1).

**FastJM** is another R package used for joint modelling of longitudinal and time-to-event data. It is beneficial for analyzing large and complex healthcare datasets, providing a range of features for efficient and scalable analysis. Bayesian methods are used to estimate model parameters, and the package offers several modelling options, including shared random effects and time-varying coefficients. Visualization and diagnostic tools are also included for model evaluation and prediction quality assessment.

**JoineRML** is an R package that allows for joint modelling of longitudinal and time-to-event data, providing a flexible and efficient approach to developing dynamic predictive models. It is beneficial for analyzing primary care big data, where careful modelling is required to extract meaningful insights. The maximum likelihood framework is used to estimate model parameters, and the package offers several modelling options, including shared random effects and time-varying coefficients. Visualization and diagnostic tools are also included to evaluate model fit and prediction quality.

**rstanarm** is an R package that provides a suite of Bayesian models for analyzing complex healthcare data, including joint models for longitudinal and survival data. It uses the Stan language to estimate model parameters, allowing for flexible and efficient modelling of complex data structures. The package offers several modelling options, including shared random effects, time-varying coefficients, and multiple correlated longitudinal outcomes. Methods for model selection and diagnostic evaluation are also included, making rstanarm a powerful tool for analyzing big healthcare data and improving patient outcomes.

While **JMbayes2, FastJM, JoineRML**, and **rstanarm** are all powerful R packages for joint modelling of longitudinal and survival data, they may not be suitable for working with big data due to computational constraints. Big data in healthcare can be extremely large and complex, requiring significant computing power and memory to analyze. While these packages offer a range of tools for efficient and accurate modelling of complex data structures, their efficiency may be limited when working with massive datasets. Thus, alternative methods such as distributed computing, parallelization, or cloud computing may be needed to analyse big data in healthcare efficiently. Nevertheless, developing these packages demonstrates the increasing need and interest in joint modelling of longitudinal and survival data in healthcare and the potential for these models to improve patient care and population health.

Our work aims to develop an automation framework for jointly modelling longitudinal and survival data. The framework will use various tools to automate the modelling process, particularly in handling large datasets in primary care.

The framework has been designed to be flexible and easy to use. Researchers can specify the desired model and data inputs, and the framework will then automatically run the analysis and generate the results. The framework can also handle missing data and complex longitudinal and survival models.

The development of this framework has made it easier for researchers to conduct joint modelling of longitudinal and survival data. This has allowed researchers better to understand the relationships between longitudinal and survival outcomes and develop more effective interventions.

The **jmBig** package provides a user-friendly interface for joint modelling and is designed to address the challenges of working with big data, such as computational efficiency and memory constraints. The package incorporates several functions for data preprocessing, modelling, and prediction, allowing for streamlined and reproducible data analysis.

By leveraging the functions, we could efficiently model complex relationships between longitudinal and survival outcomes, making personalized predictions for individuals. The automation framework facilitated joint modelling for primary care big data, where large and complex datasets require careful modelling to extract meaningful insights.

The development of this automation framework has the potential to improve patient outcomes and population health by enabling efficient and accurate analysis of big data in healthcare. The use of **jmBig** in this framework adds to the existing suite of R packages for joint modelling and further enhances the capabilities of healthcare analytics for big data.

## Methodology

Bayesian methods benefit from joint modelling of longitudinal and time-to-event data in healthcare analytics. They provide a flexible and robust framework for modelling complex relationships between multiple outcomes, such as longitudinal measurements and time-to-event outcomes. This is because Bayesian methods allow for the incorporation of prior knowledge and provide estimates of model uncertainty, which is especially important when dealing with complex and noisy healthcare data.

The relationship between longitudinal and time-to-event outcomes can be complex and nonlinear in joint modelling. Bayesian methods can handle such complex relationships by specifying flexible and complex models that can capture the underlying patterns in the data. This contrasts with traditional frequentist methods that often rely on linear or simple models, which may not adequately capture the complexity of healthcare data.

Additionally, Bayesian methods provide estimates of model uncertainty, essential when making predictions and inferences based on the model. Predictions based on models that do not account for uncertainty in healthcare analytics can lead to inaccurate and potentially harmful decisions. Bayesian methods provide a probabilistic framework for making predictions that account for uncertainty, allowing for more informed decision-making.

In this context, we need a powerful tool for efficient and accurate analysis of large-scale longitudinal and survival data in healthcare analytics. By fitting a Bayesian joint model to the data, the function can estimate the complex relationships between the outcome and predictor variables, providing more accurate and robust estimates of these relationships. The function automatically prepares the data for analysis, splits the data into smaller samples, and stores the model estimates. The function output includes model estimates, such as the mean and standard error for each parameter, and other relevant information, such as the number of observations and sample size used. Overall, the ”jmbayesBig" function provides a valuable tool for analyzing big healthcare data and improving patient outcomes and population health.

### jmbayesBig

The **jmbayesBig** function performs a Bayesian joint model analysis on longitudinal and survival data. It requires four input parameters: “dtlong" and “dtsurv", which are data frames containing the longitudinal and survival data respectively, as well as “longm" and “survm", which are formulas that specify the relationship between the outcome and predictor variables in the longitudinal and survival models. The function first checks whether the input data frames have columns named “id" and whether the “id" column is the same in both data frames. It then prepares the data for analysis by splitting it into smaller samples and creating a list of objects to store the model estimates.

The function then fits the joint model to each sample of data using the “jm" function, which implements the Bayesian joint model. The function stores the posterior samples from each sample of data and calculates the means and standard errors of the parameters across all samples.

Finally, the function returns the model estimates, including the mean and standard error for each parameter and other relevant information, such as the number of observations and the sample size used. The function returns a list object of class “jmbayesBig".

Overall, this code is used to fit a Bayesian joint model to longitudinal and survival data, which allows for the analysis of the two types of data together and can provide more accurate and robust estimates of the relationships between the outcome and predictor variables.

### jmcsBig

The jmcsBig function is a Bayesian joint model for analyzing longitudinal and survival data together. It can be used to estimate the parameters of a joint model, which is a model that takes both longitudinal data and survival data into account. The survfitJMCS function can then compute the conditional survival probabilities for specific subjects.

The code fits a joint longitudinal and survival data model using the jmcsBig function in R. The model includes a linear mixed effects model for the longitudinal data and a Cox proportional hazards model for the survival data. The output includes estimates of fixed and random effects and association parameters between the two submodels.

The jmcsBig function also provides predicted trajectories for each subject at each visit. The trajectories are accompanied by confidence intervals and prediction intervals. The output can be divided into two parts: individual trajectories and combined trajectories. The output can be used to understand trends in the data and make informed decisions.

### joinRMLBig

The joinRMLBig function is a powerful tool that can be used to analyze two types of data: longitudinal and survival data. The function can be used to estimate the parameters of a joint model, which is a model that takes both types of data into account.

The joinRMLBig function first checks that the datasets have an identifier column and that the identifier columns in both datasets are the same. It then splits the survival dataset into groups based on the sample size. The longitudinal dataset is also split into groups based on the number of individuals in each group in the corresponding survival dataset.

Next, the function fits the joint model using the ’joint’ function and stores the results for each group in a list. The function then updates the estimated coefficients and the Hessian matrix for the joint model using the results from all the groups.

Finally, the function returns a list containing the call, the fitted joint models for each group, the updated estimates for the joint model, and the sample size. This list is given to the class joinRMLBig.

**Fig. 2:**
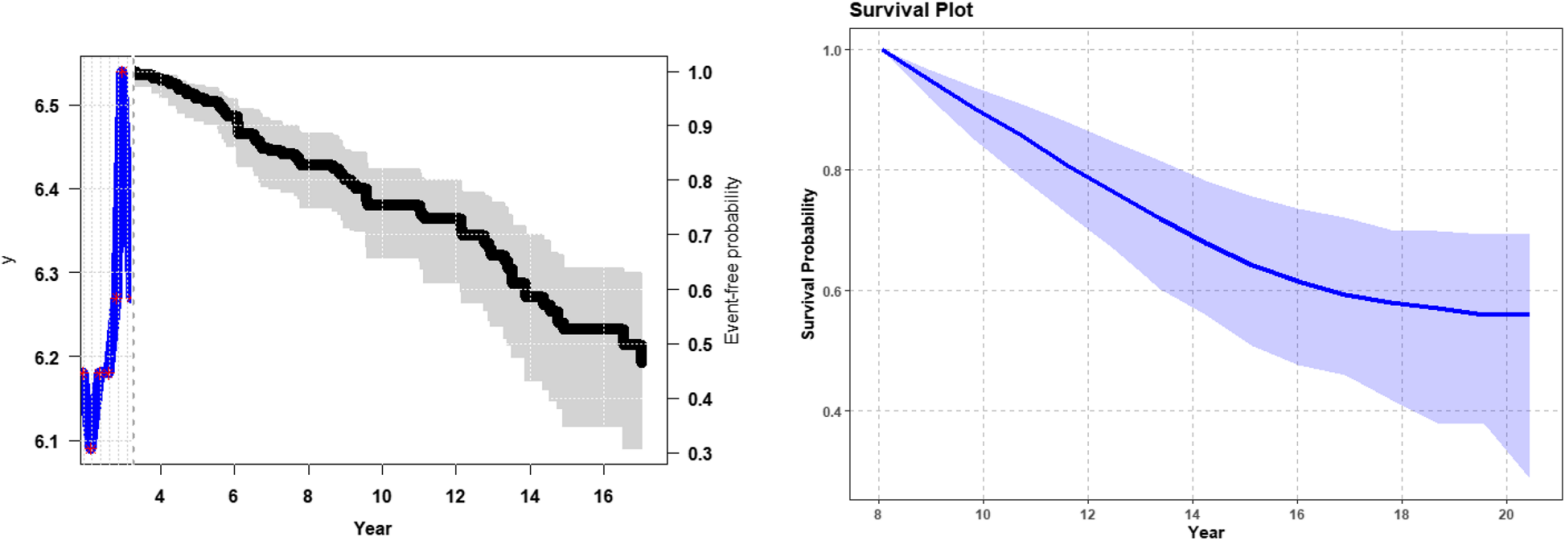
Predictions generated by the joinRMLBig algorithm utilize joint modelling techniques to bolster the precision of prognostic assessments in large-scale datasets, offering individual-level predictions for both longitudinal trajectories and survival outcomes

**Fig. 3:**
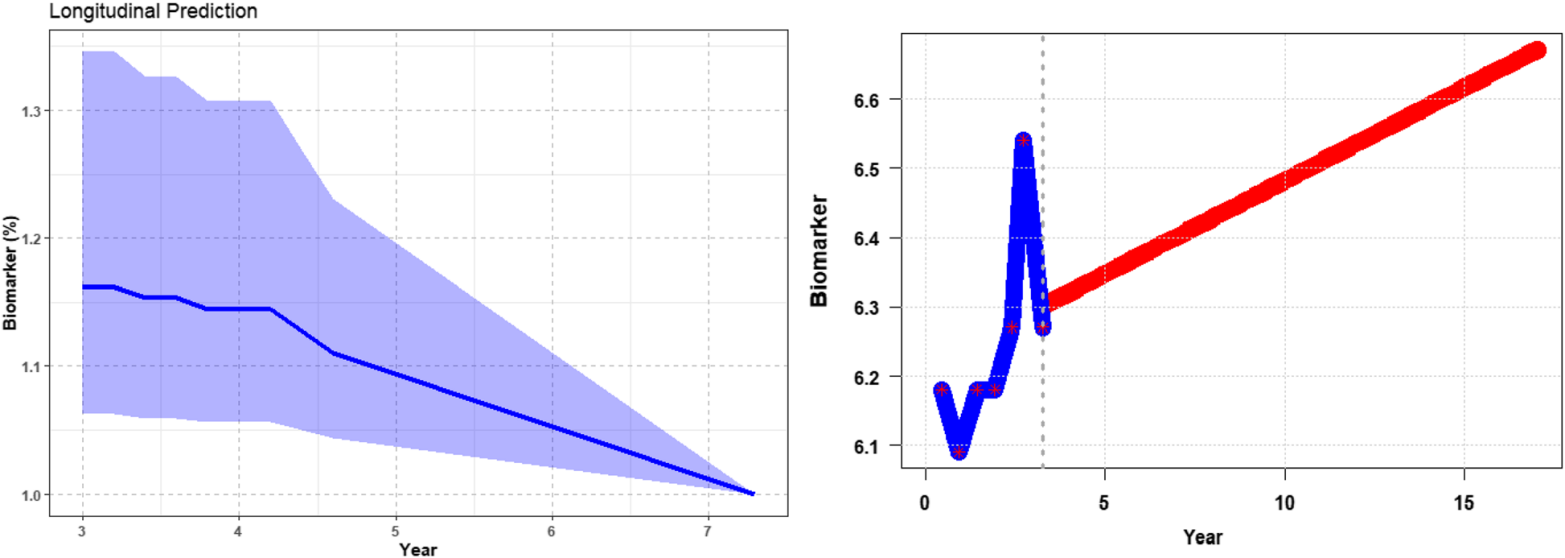
Predictions obtained on Time-to-Event Outcomes with joinRMLBig harness advanced joint modelling techniques to provide accurate prognostic insights into survival outcomes, aiding in personalized interventions for improved patient care

The results of the analysis provide a list containing three data frames: One contains predicted values for the longitudinal outcome, the second one contains predicted values for the longitudinal outcome for each cluster, and the third one contains predicted survival probabilities for each subject at each time point (Figs. 2 and 3).

The analysis results yield comprehensive insights encapsulated within four distinct figures. Firstly, the primary data frame encapsulates predicted values for the longitudinal outcome, furnishing a nuanced understanding of the trajectory and evolution of the observed variable over time (Fig. 2, left side). These predicted values serve as invaluable prognostic indicators, aiding in identifying trends, patterns, and potential areas of intervention. Secondly, within Fig. 2, the right-hand side offers a detailed depiction of survival predictions for the group of individuals. This granular presentation affords a comprehensive overview of survival outcomes, elucidating the nuanced variability among subjects. By visualizing overall survival predictions, stakeholders gain invaluable insights into the heterogeneous nature of prognostic trajectories within the cohort. This level of granularity is instrumental in optimizing resource allocation, as it allows for targeted interventions tailored to the specific needs and risks of each group of patients. With this detailed understanding, healthcare providers can allocate resources more efficiently, ensuring maximal efficacy in treatment strategies and interventions. Moreover, this approach facilitates the identification of high-risk individuals who may benefit from proactive interventions or closer monitoring, thereby enhancing overall patient care and outcomes. Lastly, Fig. 3 illuminates predicted survival probabilities for each subject at various time points based on their previously measured biomarker’s value. By offering a probabilistic outlook on survival outcomes, this data frame equips stakeholders with essential foresight, enabling pre-emptive measures to mitigate risks and enhance patient outcomes. Furthermore, the temporal dimension inherent in these survival probabilities enables the anticipation of critical junctures in the patient journey, informing timely interventions and resource allocation. Collectively, the amalgamation of these three data frames paints a comprehensive portrait of the underlying dynamics governing the observed phenomena. These insights empower stakeholders to make informed decisions, optimize resource allocation, and improve patient outcomes (see Figs. 2 and 3 for visual representations of these data frames, further enhancing comprehension and aiding in communicating findings).

### jmstanBig

The jmstanBig function can be used to estimate the parameters of a joint model, which is a model that takes both longitudinal data and survival data into account. This allows researchers to understand the relationship between the two data types and make predictions about future outcomes.

For example, a researcher might use the jmstanBig function to analyze data from a study of patients with cancer. The longitudinal data might include measurements of the patient’s tumour size at different visits. The survival data might include information about whether or not the patients have died from cancer. The jmstanBig function could be used to estimate the parameters of a joint model that predicts the patients’ tumour size and risk of death.

The ‘postTraj’ function can then be used to compute the posterior predictions for specific subjects. This means that the ‘postTraj’ function can predict the tumour size and the risk of death for individual patients.

To calculate the processing times for various functions across different dataset sizes, we leveraged a robust computer system featuring 32 GB of RAM and a 500 GB hard drive. These specifications provided ample computational resources essential for executing the analyses efficiently. Additionally, we utilized the Trusted Research Environment (TRE) on Amazon Web Services (AWS) to manage and process electronic health record (EHR) data effectively (Table 1).

**Table 1:** Processing times for different datasets using various functions

Function	Dataset Size	Processing Time (Regular) in Minutes	Processing Time (EHR) in Minutes
jmbayesBig	10 patients	5.06	11.14
	100,000 patients	19.11	29.62
	1,000,000 patients	25.65	45.23
jmcsBig	10 patients	1.02	3.07
	100,000 patients	7.43	17.11
	1,000,000 patients	13.04	29.24
joinRMLBig	10 patients	0.97	2.12
	100,000 patients	2.03	8.54
	1,000,000 patients	5.03	17.49
jmstanBig	10 patients	1.04	3.10
	100,000 patients	2.09	10.18
	1,000,000 patients	11.21	20.54

The processing times showcased in the table represent the careful allocation of computational resources for each function and dataset size combination. Notably, as the dataset size increases, the demand for computational power escalates, influencing processing times accordingly. Moreover, the juxtaposition of regular processing times with those involving EHR data underscores the heightened computational demands associated with the latter.

In summary, the synergy between our computer system’s capabilities and the utilization of appropriate software tools facilitated the extraction of valuable insights from the data analysis process outlined in the table.

**Fig. 4:**
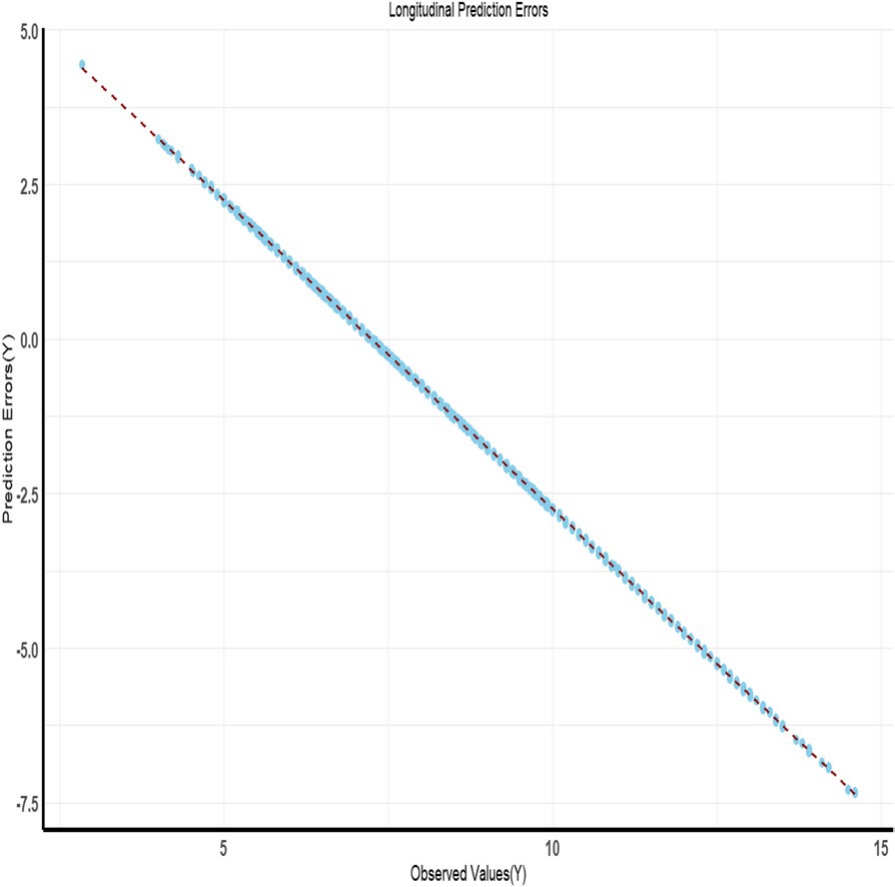
Predictions obtained on time trajectory from the longitudinal component of the joint longitudinal and time-to-event modeling approach

**Fig. 5:**
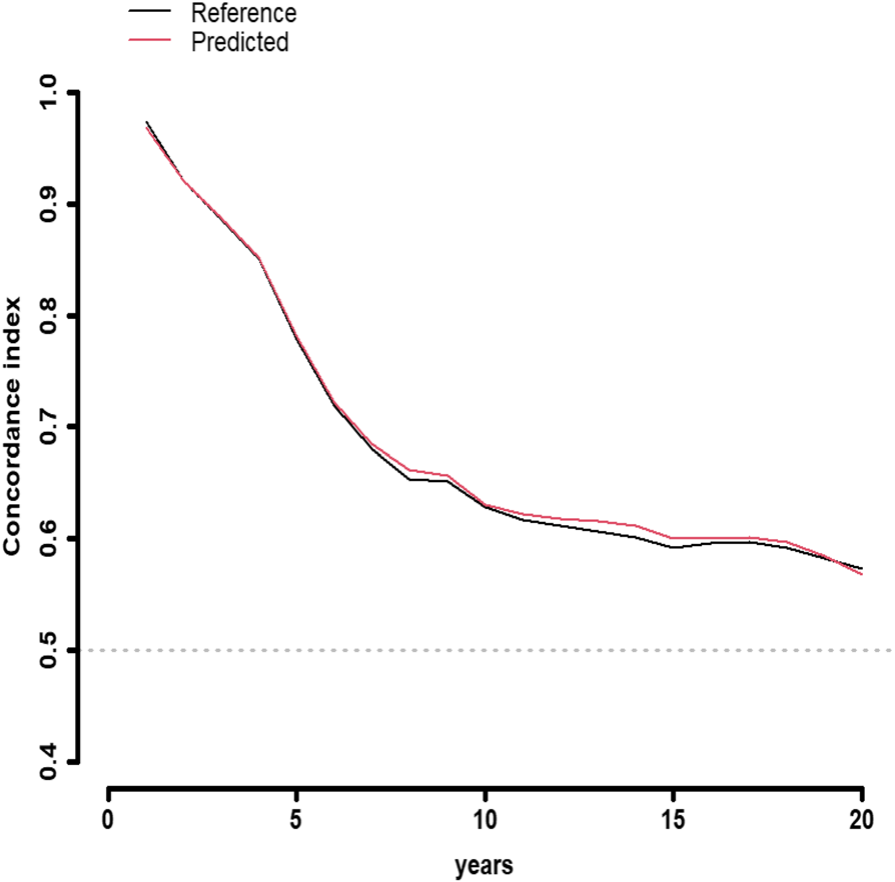
Time to event concordance index obtained from the joint longitudinal and time to event model: exploring predictive performance across time

**Fig. 6:**
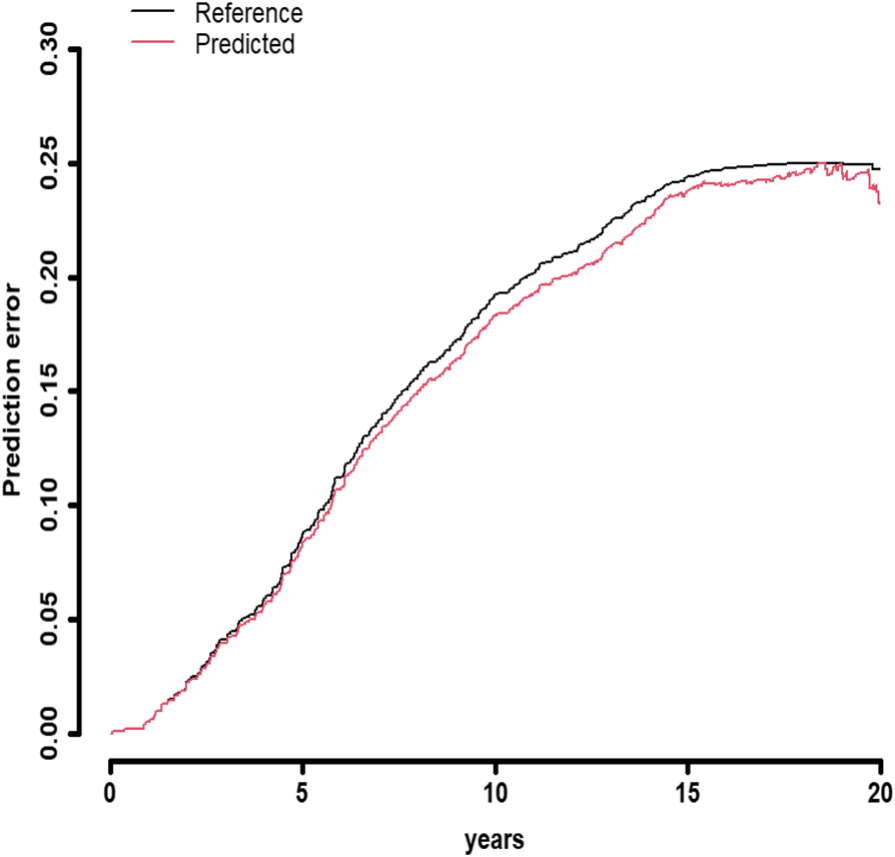
Time to event prediction error over time obtained through the model

Figure 4 illustrates predictions obtained on the time trajectory from the longitudinal component of the joint modelling approach. The concordance index derived from the joint longitudinal and time-to-event model is showcased in Fig. 5. Furthermore, Fig. 6 displays the time-to-event prediction error over time obtained through the model.

## Discussion

The use of Bayesian methods for joint modelling of longitudinal and time-to-event data in healthcare analytics is becoming increasingly popular due to their flexibility and ability to model complex relationships between multiple outcomes. This is particularly important in healthcare analytics, where the relationships between longitudinal measurements and time-to-event outcomes can be complex and nonlinear.

For example, let us say we are interested in predicting the risk of death in patients with cancer. We might have longitudinal data on the patient’s tumour size, tumour markers, other biomarkers, and time-to-event data on their survival. We could use a Bayesian joint model to model the relationship between these two data types. The model would allow us to incorporate our prior knowledge about the relationships between the variables, and it would also allow us to make personalized predictions about the risk of death for each patient.

The results of the Bayesian joint model could be presented in a graphical format, such as a Kaplan-Meier curve or a survival forest. These graphical presentations would allow us to visualize the relationship between the longitudinal measurements and the time-to-event outcome, and they would also allow us to identify patients at high risk of death.

Using Bayesian methods for joint modelling of longitudinal and time-to-event data has several advantages over traditional methods: Bayesian methods are more flexible and can model complex relationships between multiple outcomes. Bayesian methods allow us to incorporate prior knowledge about the relationships between the variables, which can improve the accuracy of the predictions. Bayesian methods can be used to make personalized predictions for each patient.

The use of Bayesian methods for joint modelling of longitudinal and time-to-event data is a promising new approach for healthcare analytics. This approach could improve the accuracy of predictions and provide personalized insights for patients and clinicians. Bayesian methods allow for the incorporation of prior knowledge and provide estimates of model uncertainty, which is crucial when dealing with complex and noisy healthcare data. Predictions based on models that do not account for uncertainty can lead to inaccurate and potentially harmful decisions. Bayesian methods provide a probabilistic framework for making predictions that account for uncertainty, allowing for more informed decision-making.

The **jmbayesBig** function provides a valuable tool for analyzing big healthcare data and improving patient outcomes and population health. It allows for the efficient and accurate analysis of large-scale longitudinal and survival data by fitting a Bayesian joint model to the data. The function automatically prepares the data for analysis, splits the data into smaller samples, and stores the model estimates. The function output includes model estimates, such as each parameter’s mean and standard error, and other relevant information, such as the number of observations and sample size used.

Similarly, the **jmcsBig** function is used to fit a joint model for longitudinal and survival data. It estimates fixed and random effects and association parameters between the longitudinal and survival submodels. The function also provides a ’postTraj’ function, which displays the predicted trajectories for each subject at each visit, along with fitted values and confidence intervals for the predicted values. This information can help make informed decisions and understand the trends in the data.

Overall, joint modelling in healthcare analytics can provide more accurate and robust estimates of the relationships between the outcome and predictor variables, leading to improved patient outcomes and population health. Bayesian methods and the functions discussed in this text are powerful tools for efficient and accurate analysis of large-scale healthcare data. However, it is essential to note that additional context about the research question, study design, and specific statistical methods is necessary to draw meaningful conclusions from the output. Running the code on a dataset containing 1000 unique individuals with multiple visit observations and time to death recorded at various time points revealed varying durations for analysis. Specifically, for the jmBayesBIG package, the analysis took approximately 2.3 minutes. joinrmlBIG required 1.2 minutes, while jmstanBIG and jmcsBIG each took around 2.72 and 1.2 minutes, respectively.

The dashboard for JMbig is readily accessible at https://atanuucsi.shinyapps.io/jmapp. This intuitive platform empowers users to upload their data effortlessly and efficiently conduct detailed analyses. With its user-friendly interface, individuals from various backgrounds can navigate the features seamlessly, gaining valuable insights into their data. Whether exploring individual-specific predictions or evaluating model performance, this dashboard is valuable for researchers, clinicians, and data analysts. Historically, these two processes have been analyzed separately. For example, time-to-event data is typically modelled using hazard-based regression models. These include the Cox proportional hazards (PH) model [12], accelerated failure time (AFT) models [13], accelerated hazard (AH) models [14], and general hazard (GH) structures [15].

The longitudinal process is typically modelled using generalized linear mixed models (GLMMs) [16]. GLMMs are a powerful tool for incorporating the information in both the longitudinal and survival processes.

Joint modelling of longitudinal and survival processes has been extensively discussed in recent literature [10, 17, 18]. A common strategy in joint models is to link the survival and longitudinal processes by including shared parameters on the models for the covariates. This allows for incorporating several statistical modelling tools already available in the literature, such as using flexible parametric models using splines for modelling the hazard or the cumulative hazard functions [19]. The longitudinal process can be modelled using any techniques developed for GLMMs.

Our package is validated through internal testing, preliminary real-world datasets, and ongoing external validation and benchmarking. Identified limitations include handling missing data, computational efficiency for large datasets, limited covariate support, and the need for user-provided initial values. Planned enhancements include customizable graphical outputs, improved visualization tools, and additional documentation for easier user adoption. We plan to enhance data imputation, optimize algorithm efficiency, expand support for diverse data types and flexible modelling, and conduct a comprehensive validation study with external datasets. User feedback will be incorporated to refine and improve the package continually. These efforts aim to provide a clear overview of our package’s current state and ongoing development, enhancing its utility and practicality.

## Conclusion

Joint modelling of longitudinal and survival data using Bayesian methods is a powerful and flexible approach for analyzing complex healthcare data. By allowing for the incorporation of prior knowledge and accounting for uncertainty, Bayesian methods can provide more accurate and robust estimates of the relationships between multiple outcomes, such as longitudinal measurements and time-to-event outcomes. In particular, joint modelling is essential when dealing with big healthcare data, as it can help researchers better understand the complex relationships between various predictors and outcomes. With the increasing availability of large-scale longitudinal and survival data in healthcare, there is a growing need for efficient and accurate analysis tools. The **jmbayesBig, jmcsBig, jmstanBig,** and **joinRMLBig** functions are all examples of powerful tools that allow for the efficient and accurate analysis of big healthcare data. These functions can handle large datasets, split them into smaller samples, and provide accurate estimates of the parameters of interest. Overall, joint modelling of longitudinal and survival data provides a valuable approach to healthcare analytics, particularly in the context of big data. By incorporating prior knowledge, accounting for uncertainty, and modelling complex relationships between multiple outcomes, joint modelling can help researchers and healthcare professionals better understand the complex nature of healthcare data and make more informed decisions to improve patient outcomes and population health.

## Data Availability

The datasets generated and/or analysed during the current study are available in the ‘jmBIG’ package available in CRAN. [NAME] repository, [https://cran.r-project.org/web/packages/jmBIG/index.html].
